# The PTGS2/COX2-PGE_2_ signaling cascade in inflammation: Pro or anti? A case study with type 1 diabetes mellitus

**DOI:** 10.7150/ijbs.86492

**Published:** 2023-08-06

**Authors:** Eugenia Martín-Vázquez, Nadia Cobo-Vuilleumier, Livia López-Noriega, Petra I. Lorenzo, Benoit R. Gauthier

**Affiliations:** 1 Andalusian Center of Molecular Biology and Regenerative Medicine CABIMER, Junta de Andalucia-University of Pablo de Olavide-University of Seville-CSIC, Seville, Spain.; 2 Centro de Investigacion Biomedica en Red de Diabetes y Enfermedades Metabolicas Asociadas (CIBERDEM), Madrid, Spain.

**Keywords:** Beta cells, Cyclooxygenases, Inflammation, Pancreatic Islets, Prostaglandin, Type 1 Diabetes Mellitus

## Abstract

Prostaglandins are lipid mediators involved in physiological processes, such as constriction or dilation of blood vessels, but also pathophysiological processes, which include inflammation, pain and fever. They are produced by almost all cell types in the organism by activation of Prostaglandin endoperoxide synthases/Cyclooxygenases. The inducible Prostaglandin Endoperoxide Synthase 2/Cyclooxygenase 2 (PTGS2/COX2) plays an important role in pathologies associated with inflammatory signaling. The main product derived from *PTGS2/COX2* expression and activation is Prostaglandin E_2_ (PGE_2_), which promotes a wide variety of tissue-specific effects, pending environmental inputs. One of the major sources of PGE_2_ are infiltrating inflammatory cells - the production of this molecule increases drastically in damaged tissues. Immune infiltration is a hallmark of type 1 diabetes mellitus, a multifactorial disease that leads to autoimmune-mediated pancreatic beta cell destruction. Controversial effects for the *PTGS2/COX2*-PGE_2_ signaling cascade in pancreatic islet cells subjected to diabetogenic conditions have been reported, allocating PGE_2_ as both, cause and consequence of inflammation. Herein, we review the main effects of this molecular pathway in a tissue-specific manner, with a special emphasis on beta cell mass protection/destruction and its potential role in the prevention or development of T1DM. We also discuss strategies to target this pathway for future therapies.

## 1. Introduction

Type 1 Diabetes Mellitus (T1DM) is a disease caused by the selective destruction of pancreatic islet beta cells by aberrant activation of the immune system, characterized by a subsequent unresolved proinflammatory status within the pancreas 1, 2. To date, no effective therapies have been developed to cure this autoimmune disorder, which indeed, apart from the beta cell death and subsequent lack of insulin, leads to long-term complications that substantially impact on life quality and shorten life expectancy 2. Type 1 Diabetes Mellitus is a T cell-mediated autoimmune disease - the main cause is an imbalance between T regulatory and autoreactive CD4^+^ and CD8^+^ T effector cells, which make them react specifically against pancreatic islet-associated self-antigens, leading to beta cell mass destruction. In this context, infiltrating macrophages and T effector cells secrete different pro-inflammatory cytokines, promoting a chronic pro-inflammatory microenvironment within the pancreas 3-5. However, we and others have reported that some level of inflammation is beneficial for the regeneration of beta cell mass as it induces a dialogue between pancreatic and immune cells aimed at protecting beta cells 6, 7. This fact could explain the failure of potential reported treatments for T1DM based on either blocking the immune attack or aimed at the preservation of the beta cell mass 8. Based on this, we have recently proposed a model in which T1DM can be conceived as an 'unresolved wound healing process', where the pro-inflammatory phase persists and cannot be resolved 2. According to this model, pathways that may modulate inflammation, instead of supressing the immune system, may be promising therapeutic targets for T1DM.

### 1.1 Prostaglandins: General overview on their physiological roles

Cyclooxygenases (COXs) are the enzymes that catalyse the first rate-limiting step in prostaglandins (PGs) synthesis from arachidonic acid (AA). There are 2 main COXs isoforms; the constitutive cyclooxygenase 1, also known as (*a.k.a.*) Prostaglandin Endoperoxidase Synthase 1 (COX1/PTGS1) widely expressed in most tissues, and the stress-inducible cyclooxygenase 2, *a.k.a.* Prostaglandin Endoperoxidase Synthase 2 (COX2/PTGS2) normally expressed at very low levels under physiological conditions 9, 10. PTGS1/COX1 is a housekeeping enzyme - it is required for maintaining basal PGs levels for tissue/cell homeostasis 9. On the other hand, PTGS2/COX2 is tightly regulated, and its expression and activation are directly induced by pro-inflammatory cytokines and growth factors that activate intracellular inflammation-related pathways 11. Prostaglandin E_2_ (PGE_2_) is the main biologically-active PG-derived from the metabolization of AA 12 and it is mainly involved in inflammation-related processes 13. There is a third reported isoform, Cyclooxygenase 3/ Prostaglandin Endoperoxide Synthase 3 (COX3/PTGS3), although this one has been barely studied, as it is a splice variant of PTGS1/COX1, encoding a truncated protein lacking enzymatic activity 14.

Focusing on PTGS2/COX2, overwhelming opposite effects have been reported for this molecular pathway: Due to its expression and activation during inflammation, the PTGS2/COX2-PGE_2_ signalling axis has been widely thought to be a driver of inflammation, induced as a direct cause of it 10, 15. However, subsequent studies reported that both, endogenous production or repletion with PGE_2_ analogues restored tissue homeostasis upon stress or proinflammatory conditions 16, 17. As such, the probable involvement of PTGS2/COX2-PGE_2_ in the resolution phase of the inflammatory process points at this signalling pathway as a potential therapeutic target for pathologies displaying high levels of inflammation. We presently review the effects of PTGS2/COX2-PGE_2_ cascade in different inflammatory contexts, focusing on pancreatic beta cells subjected to T1DM conditions and discuss its potential exploitation as therapeutic target for the autoimmune pathology.

### 1.2 Biosynthesis of PGE_2_ and its downstream signalling pathways

**Figure 1:**
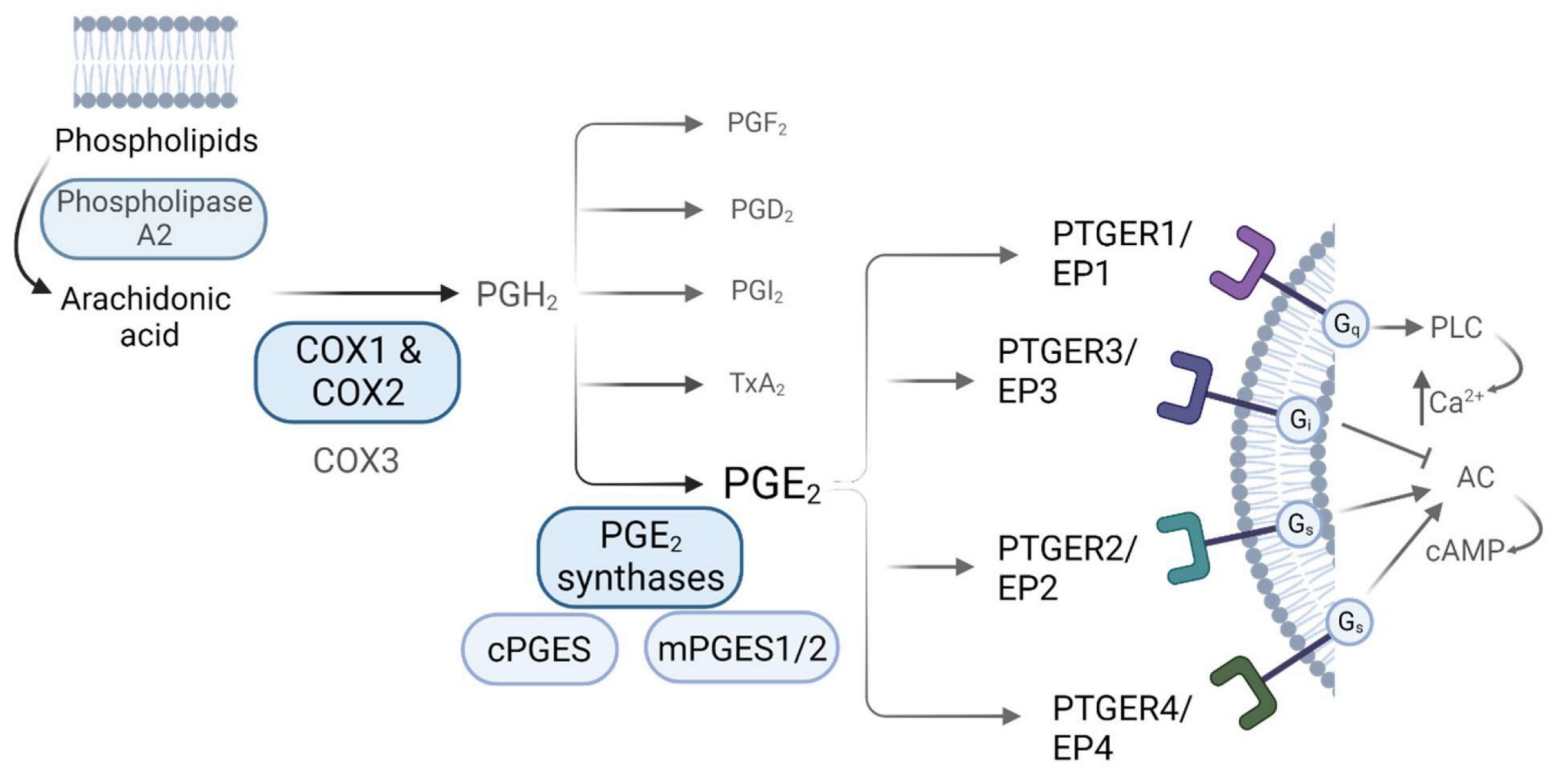
** PGE_2_ synthesis via the Arachidonic Acid pathway**. Biosynthesis of prostaglandins. AA is liberated from membrane phospholipids by the action of Phospholipase A_2_, that is then converted into PGH_2_ by the action of the Cyclooxygenases, a rate-limiting step for prostaglandin biosynthesis. Prostaglandin H_2_ (PGH_2_) is the unstable precursor of Prostaglandin F_2_ (PGF_2_), Prostaglandin D_2_ (PGD_2_), Prostaglandin I_2_ (PGI_2_), Thromboxane A_2_ (TxA_2_) and Prostaglandin E_2_ (PGE_2_). PGE_2_ receptors and their related downstream signaling are also shown.

Prostaglandins are synthetized via the arachidonic acid (AA) pathway. Arachidonic acid is liberated from membrane phospholipids by the action of phospholipase A_2_ (PLA_2_), and then converted into the unstable precursor Prostaglandin H_2_ (PGH_2_) 13. This conversion constitutes the first rate-limiting step in PGs synthesis, and it is performed by the action of the COXs 9, 10. Once PGH_2_ is formed, it is converted into different prostaglandins by tissue-specific Prostaglandin Synthases (PGS). PGE_2_ can be produced by the action of both, cytosolic Prostaglandin E Synthases (cPGES) and microsomal Prostaglandin E Synthases1/2 (mPGES1/2) 13. COXs-derived PGs mediate their actions via binding to receptors coupled to different G (guanine nucleotide-binding) proteins, denoted as G-protein coupled receptors (GPCRs), which are widely expressed throughout the body and whose activation induce distinct intracellular signalling cascades. Regarding PGE_2_, there are 4 known GPCR subtypes: PTGER1 (*a.k.a.* EP1) that employs Gq (G protein heterotrimeric, that activates beta isoforms of phospholipase C (PLC) and induces calcium mobilization), PTGER3 (*a.k.a.* EP3) that employs Gi (G inhibitory protein, which decreases the cAMP levels by inhibiting adenylyl cyclase (AC) activity), and PTGER2 and PTGER4 (*a.k.a.* EP2 and EP4), which both utilize Gs (G stimulatory protein, that stimulates the cAMP dependent pathway by activating AC) (**Figure 1**) 11, 18. The affinity of PGE_2_ for these receptors, along with the expression levels of each of them in a specific environment, leads to different physiological outcomes. Both PTGER3/EP3 and PTGER4/EP4 possess the highest affinities for the prostaglandin (dissociation constant (Kd) values of 0.33-2.9nM and 0.59-1.27nM, respectively) while PTGER1/EP1 exhibits the lowest affinity (Kd of 16-25nM). The affinity of PTGER2/EP2 for PGE_2_ differs between species - the rat PTGER2/EP2 exhibits higher affinity for PGE_2_ as compared to mouse and human ones 18.

## 2. PTGS2/COX2-PGE_2_ role upon inflammatory signaling: tissue-specific crosstalk with immune cells

Despite widely foreseen as an inducible gene, exceptions for constitutive PTGS2/COX2 expression include testes 19, kidney - macula densa cells 20 and the central nervous system (CNS) 21, 22. For instance, in the brain, PTGS2/COX2 expression was shown in specific subsets of neurons dispersed throughout the tissue, where it participates in synaptic activity and memory consolidation. Indeed, aberrant upregulation of *Ptgs2/Cox2* expression in vascular endothelial cells from the CNS has been linked to neurotoxicity and chronic neurodegenerative processes in rats treated with Carrageenan, a well-known acute inflammation model 23, 24.

Several studies have attempted to address whether PTGS2/COX2 expression, and specially PGE_2_ production, convey either beneficial or detrimental effects using different models of inflammation. Initial studies performed in Caco-2 colorectal cells treated with exogenous PGE_2_ showed that the lipid mediator was able to upregulate proinflammatory pathways such as NF-κB 25. Remarkably, PTGS2/COX2 exhibits a specific NF-κB binding site in its promoter region, therefore, a feedback loop involving PTGS2/COX2 expression and further endogenous PGE_2_ synthesis upon proinflammatory conditions could be taking place, leading to increased NF-κB signalling 26. However, subsequent studies reported overwhelming opposite effects. For instance, repletion with PGE_2_ analogues has been reported to restore homeostasis during inflammation *in vivo*, in a mouse model of immune arthritis 16. This phenotype appeared to be facilitated by PGE_2_-mediated inhibition of NF-κB, therefore decreasing the pro inflammatory cytokines production 27. This event reveals a very interesting therapeutic loop that could resolve inflammation and restore tissue homeostasis: PGE_2_ production may induce NF-κB, but later on, as it is necessary to resolve the inflammatory process, the final outcome is decreased NF-κB activation. Indeed, it has been widely reported that conventional nonsteroidal anti-inflammatory drugs (NSAIDs), the most common inhibitors of PTGS2/COX2, exhibit palliative (rather than curative) effects 28, 29, a fact that could be explained by the impossibility to resolve inflammation upon PTGS2/COX2 inhibition.

Focusing on the immune system, PGE_2_ treatment has been shown to alter macrophage polarization and metabolism towards an M2/anti-inflammatory phenotype. This event has been observed in white adipose tissue, in the *ob/ob* mouse model of obesity 30. PGE_2_ synthesis was also shown to be upregulated in human T-cells as an early inducible gene upon T-cell activation 31 and to exert opposite effects in a 'concentration-dependent' manner, leading to different scenarios of homeostasis and inflammation during T-cell responses. At high concentration, PGE_2_ was shown to promote an anti-inflammatory environment by suppressing the T helper 1 (Th1) subpopulation while activating the T helper 2 (Th2) subset of T-cells 32, and by inducing the differentiation of FOXP3^+^CD4^+^CD25^+^ adaptive regulatory T-cells that inhibit T-cell effector responses. The aforementioned suppressive properties were conveyed via binding to PTGER2/EP2 and PTGER4/EP4, the PTGERs expressed by T-cells 33. In contrast, low concentrations of PGE_2_ have been shown to induce Th1 differentiation patterns, conveying a proinflammatory phenotype, via PTGER4/EP4 signaling 34. These findings highlight the tight regulation of the PTGS2/COX2-PGE_2_ signaling cascade that can lead to opposite scenarios in contexts of inflammation.

**Figure 2:**
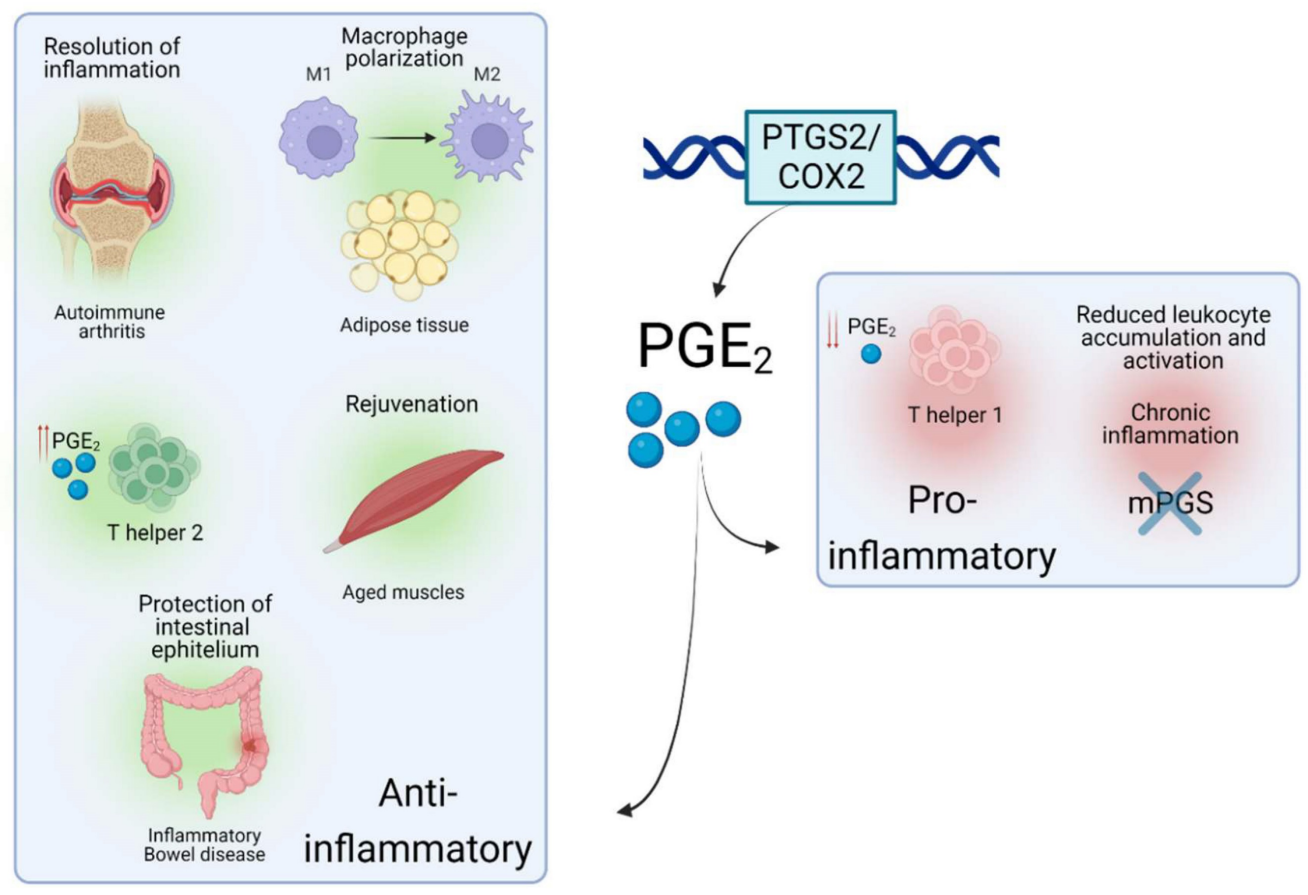
** Roles of PTGS2/COX2-PGE_2_ in different inflammatory situations.** Schematic view of the role and effects of PTGS2/COX2-PGE_2_ upon different inflammation-related environments.

Interestingly, a recent study reported that PGE_2_ levels were decreased in muscles upon aging, a progressive and degenerative multifactorial condition, finely intertwined with inflammatory responses. In mice, depletion of PGE_2_ directly correlated with muscle wasting, while restoration of PGE_2_ levels via inhibition of the PGE_2_-degrading enzyme (15- PGDH) ameliorated the effect of age-related processes in muscle. This was achieved via a complete 'rejuvenation' of mitochondrial function and downregulation of the ubiquitin and TGFβ pathways. Interestingly, a tendency towards upregulated 15- PGDH enzymatic activity and decreased levels of PGE_2_ was observed in tissue-resident macrophages 35 which led the authors to conclude that these cells are a major site of PGE_2_ degradation and as such, drivers of the dysfunction of the aged environment. This study highlights a potential paracrine interaction between immune and tissue-specific cell types via PTGS2/COX2-PGE_2_ signaling, which could be targeted in inflammation-related pathologies. Such crosstalk via PTGS2/COX2-PGE_2_ has been documented in several cellular contexts. A subtype of tissue-resident innate lymphoid cells was recently shown to protect the intestinal epithelium from Tumour Necrosis Factor (TNF)-induced cell death by producing a heparin-binding epidermal growth factor promoted by PGE_2_ production in inflammatory bowel disease, a chronic inflammation-related disorder of the intestinal tract 36. Similarly, in a mouse model of chronic inflammation, a novel interaction between the pro-inflammatory Interleukin-17 (IL-17) and the microsomal PGE_2_ synthase (**Figure 1**) generated by macrophages was shown to reduce leukocyte infiltration and myeloperoxidase activity 37. These data substantiate that the PTGS2/COX2-PGE_2_ signalling promotes different immune-related effects, which mainly depends on the context of the local proinflammatory injury and levels of PGE_2_. In some cases, PGE_2_ would be involved in paracrine communication within different cell types, mainly immune and tissue-specific cells - a summary of several roles of the PTGS2/COX2-PGE_2_ pathway in different inflammatory scenarios is shown in **Figure 2**. In this line, some studies have emerged attempting to determine the role of this signalling pathway upon the immune-tissue specific crosstalk that characterizes autoimmune-related diseases, such as T1DM 2.

## 3. PTGS2/COX2-PGE_2_; beneficial or detrimental for type 1 diabetes development?

The specific impact of PTGS2/COX2-PGE_2_ signalling on beta cell health and functions upon diabetes features has not been fully established, yet a few studies aimed at dissecting the role of this cascade within this context have been performed. Interestingly, a study dating back to 1876, demonstrated that administration of aspirin, a potent PTGS/COX inhibitor, was proficient in normalizing blood glucose and reverting diabetes 38. These anti-diabetic effects were confirmed 100 years later in individuals with type 2 diabetes mellitus (T2DM): high-dose aspirin therapy reduced fasting glucose levels in blood, decreased total cholesterol and triglycerides and improved peripheral insulin-stimulated glucose uptake 39. However, rather than depending exclusively on PTGS/COX inhibition, the authors highlighted that the treatment with aspirin/salicylates blunted the activity of the serine kinase IKKbeta 40, which plays a key role in the pathogenesis of insulin resistance 41. In the same line, Goldfine and colleagues confirmed the robust anti-hyperglycaemic effect of a salicylate-derivative, salsalate (a dimeric pro-drug of salicylate), in individuals with T2DM 42. Whether, these beneficial effects of aspirin/salsalate are mediated via inhibition of PTGS/COX, rather than the well-defined target IKKbeta, remains to be determined. Interestingly, such studies have not been reported for T1DM. In contrast, other studies have linked the expression of the PTGS2/COX2 isoform in beta cells to the development of pathological processes in diabetes, e.g., exposure of mouse and human islets to hyperglycaemic conditions, in culture in case of human, and *in vivo*, in diabetic mice, induced PTGS2/COX2 expression and activation 43, 44. Remarkably, *Ptgs2/Cox2*-induced expression and PGE_2_ production in islet beta cells was shown to promote hyperglycaemia *in vivo*, in transgenic mice overexpressing *Ptgs2/Cox2* and Prostaglandin E Synthase (*Pges*) in beta cells under the rat insulin promoter (RIP). This effect was mainly linked to reduced proliferation of beta cells 45. These findings raised the question whether hyperglycaemia was the cause, or just a consequence of PTGS2/COX2 expression, which in any case, was apparently linked to detrimental effects. In this regard, mice treated with a high dose of streptozotocin (STZ, a chemically-induced diabetes model), displayed an increase in *Ptgs2/Cox2* expression in whole pancreas and isolated islets, but strikingly, these authors found that constitutive *Ptgs2/Cox2* knock out animals displayed significantly a higher incidence of hyperglycaemia, both after a single high dose and after several low doses of STZ 46.

In line with the reported role of PTGS2/COX2 activation upon inflammation, sometimes beneficial/anti-inflammatory, and some others detrimental/pro-inflammatory, these results pinpointed to a controversy on the impact of PTGS2/COX2 expression in the pathogenesis of diabetes, especially in beta cells. Indeed, when moving to the immune field, PTGS2/COX2 expression has been detected in monocyte-cell cultures from diabetic patients, and linked to an increase in advanced glycated end products and their receptor (AGE-RAGE) expression 47. Advanced glycated end product deposition is known to take place in diabetic blood vessels, driven by hyperglycaemia and exacerbating proinflammatory signaling 47, 48. Similarly, microarray analyses performed in peripheral blood mononuclear cells (PBMCs) from individuals with T1DM revealed an upregulation of PTGS2/COX2 expression compared to control individuals 49. Furthermore, other studies showed that monocytes from individuals at high risk of T1DM development as well as from individuals with established T1DM expressed higher levels of PTGS2/COX2 when compared to monocytes from control normoglycemic individuals 50, 51.

Taken together, these studies indicate that PTGS2/COX2 expression is likely involved in the pathogenesis of diabetes, taking part in the persistent proinflammatory status and potentially leading to detrimental consequences. Nevertheless, in the past decade, further research and several discoveries led to a re-evaluation on the concept that PTGS2/COX2 is a factor contributing to the pathogenesis and the proinflammatory environment in a diabetic context.

## 4. The effect of the PTGS2/COX2-PGE_2_ signaling is contingent on the PTGERs/EPs in islet beta cells

As previously mentioned, and contrary to previous evidence, *Ptgs2/Cox2* was subsequently shown to partially protect mouse islets from STZ-induced diabetogenic toxicity *in vivo*, using *Ptgs2/Cox2-*deficient mouse models. While no differences were reported between *Ptgs1/Cox1* knock out and control mice in terms of blood glucose levels after short-term STZ administration, *Ptgs2/Cox2* knock out animals exhibited a stronger induction of hyperglycaemia. This phenotype was further confirmed using a pharmacological approach, a selective PTGS2/COX2 inhibitor (SC-236) that also increased hyperglycaemia susceptibility in wild type (WT) animals 46. Moreover, PTGS2/COX2 was also linked to enhanced mouse and human islet survival in the presence of pro-inflammatory cytokines *ex vivo* 52. These beneficial effects appeared to be mediated by PGE_2_ interaction with one or multiple PTGERs/EPs and their subsequent activation, leading to different physiological outcomes 46, 52. This would be a plausible explanation of discrepancies reported in terms of the role of this pathway during inflammation. Both PTGER3/EP3 and PTGER4/EP4 have received the most attention, as they exhibit the highest affinities for PGE_2_ 18 and remarkably, in terms of immunomodulation, opposite effects have been reported for these two receptors: In phagocytes from different tissues, PTGER3/EP3 activation has been linked to immunostimulatory signalling, while PTGER4/EP4 activation was linked to the opposite effect, conveying an immunosuppressive environment 53.

**Figure 3:**
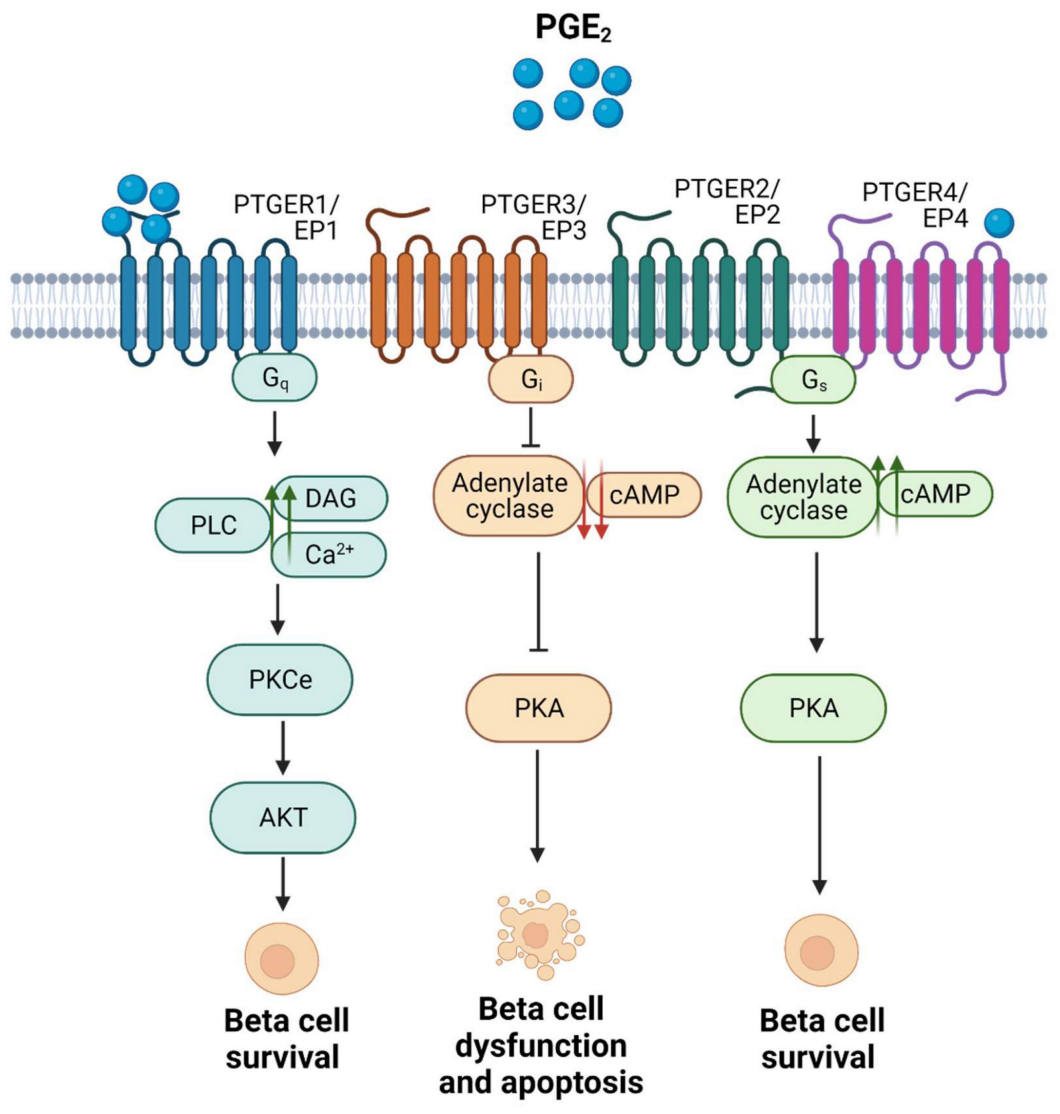
** Summary of the main signaling pathways activated upon the 4 subtypes of PTGERs/EPs and the physiological outcomes in terms of beta cell survival.** Proposed model of shift in receptor selectivity upon elevated production of PGE_2_.

Focusing on the pancreatic niche, expression of PTGER3/EP3 was shown to be increased in pancreatic islets from individuals with T2DM, as well as in MIN6 cells exposed to palmitate. Activation of this receptor in beta cells by PGE_2_ binding was associated with beta cell dysfunction and apoptosis 52, 54. In addition, PTGER3/EP3 activation has been linked to inhibition of glucose stimulated insulin secretion (GSIS) caused by a decrease in cAMP levels, via inhibition of adenylyl cyclase (AC) activity (**Figure 3**). The latter was shown in a glucose-responsive beta cell line (HIT cells), and subsequently in rodent islets 55, 56. These detrimental effects have been confirmed in functional studies, e.g., blockade of PTGER3/EP3 in the *db/db* mouse model (non-obese diabetic mice) prevented beta cell death and enhanced beta cell mass and identity. The authors found that the systemic PTGER3/EP3 antagonism promoted an increase in beta cell proliferation, restored normal islet morphology and promoted expression of genes involved in beta cell identity and function. Moreover, they encountered activation of the Nrf2 antioxidant pathway and partial restoration of GLP-1R protein expression, which was undetectable in *db/db* islets from untreated mice 57. Since GLP-1 agonism based therapies have been a major focus of promising strategies for individuals with T2DM, albeit not always showing high efficiency 58, this study supports that a combinatory treatment could provide beneficial outcomes.

In contrast, activation of PTGER4/EP4 has been reported to enhance beta cell survival in mouse and human islets *ex vivo*, in response to cytokines 59, potentially via downstream activation of the Phospho Kinase A (PKA) signalling pathway, which promotes an increase in the levels of cAMP by activating AC and subsequent phosphorylation of the cAMP response element binding (CREB) factor (**Figure 3**) 53, 59.

These data clearly show a major role for PTGS2/COX2-PGE_2_ in the beta cell physiology and pathophysiology, which can be detrimental or beneficial for beta cell function and survival depending on the PTGER(s)/EP(s) mediating the effect.

As the various outputs of this pathway seem to mainly depend on the expression levels and activity of the 4 PTGERs/EPs, interventions such as blockade of the proapoptotic PTGER3/EP3 or activation of the anti-apoptotic PTGER4/EP4 have been explored in terms of conveying protection of the beta cell mass against cytokines-induced apoptosis 57, 59. However, the specific impact of these receptors, along with those towards which PGE_2_ exhibits less affinity, has been barely studied in T1DM pathogenesis. Both PTGER1/EP1 and PTGER2/EP2 exhibit lower affinities for the prostaglandin, therefore, research focusing on them under diabetogenic conditions has been scarcely performed. However, *in vivo* global *Ptger2/Ep2* knock out was shown to exacerbate STZ pathology, remarkably, only upon PTGER4/EP4 pharmacological inactivation, showing potential pro survival properties for this receptor 46. Similarly, our group has recently shown anti-apoptotic effects via inhibition of the intrinsic apoptosis pathway mediated via PTGER1/EP1 signaling *ex vivo,* in mouse islets challenged with pro inflammatory cytokines. Our study showed a major increase in PGE_2_ synthesis in mouse islets after cytokines exposure, and an unexpected massive production of the prostaglandin when combining the cytokines treatment with BL001 17, a small chemical agonist of the nuclear receptor Liver Receptor Homolog-1 (LRH1/NR5A2) developed by our group as a potential anti-diabetic drug 6. We uncovered a new pro-survival effect of PGE_2_ mainly mediated by PTGER1/EP1 upon BL001 treatment 17, instead of PTGER4/EP4 as reported by other studies performed both in T2DM 59 and T1DM models 46. Indeed, when blocking PTGER4/EP4, we still reported protection from cytokines-induced apoptosis upon BL001 treatment, showing that the latter was not the receptor mediating the protection of LRH-1/NR5A2 activation 17. Similar to the findings of Bosma and colleagues 57, our results shed light on potentially combinatory treatments, i.e., BL001+PTGER1/EP1 agonist(s), aimed at prompting additional antidiabetic benefits.

As mentioned before, PTGER1/EP1 activation has been reported to increase diacylglycerol (DAG) production and Ca^2+^ levels, and to promote protein kinase B (AKT) phosphorylation via protein kinase C (PKC) activation (**Figure 3**) 11, 18. Remarkably, phospho-AKT-mediated signalling has been associated with beta cell survival and proliferation 60, 61, and PKC activators such as yeRACK improve islet survival during their isolation procedure, as well as their functionality after transplantation in mice 62. Since PGE_2_ exhibits the lowest affinity for PTGER1/EP1 and the highest for PTGER4/EP4 18, we proposed that high concentrations of PGE_2_ upon a post transcriptional regulation of *Ptgs2/Cox2* may favour a shift in pro-survival receptors selectivity, from PTGER4/EP4 towards PTGER1/EP1 (**Figure 3**) 17. This hypothesis needs to be further confirmed. Noteworthy, PGE_2_ has been also correlated with the expression of inhibitor-of-apoptosis proteins such as SURVIVIN through PTGER1/EP1 receptor signaling in a cancer context 63. Taken together, these studies emphasise on the high complexity of the interaction network between *PTGS2/COX2*-PGE_2_ and the various PTGERs/EPs under proinflammatory/stress conditions and other environmental factors.

## 5. Concluding remarks

Herein, we discuss the complex *PTGS2/COX2*-PGE_2_ signaling cascade upon different inflammatory scenarios. We focused on its role in inflammation and on the crosstalk between the immune system and different tissue-specific cell types, especially on pancreatic beta cells under diabetes pathophysiology. We describe reported phenotypes observed upon activation of the different PTGERs/EPs in immune cells and with special emphasis, in beta cells, which further supports the promiscuity of this signaling axis, and conclude that fine-tunning PGE_2_ production and promoting activation/blocking of the different receptors would be mandatory interventions to achieve a desirable output during stress or inflammation. Moreover, we emphasize on the fact that it is essential to consider the effect of all the 4 PTGERs/EPs, including the ones towards which the prostaglandin exhibits less affinity, such as PTGER1/EP1 17. Since* PTGS2/COX2*-PGE_2_ signaling cascade has been widely shown to play a fundamental role within immune cells 30, 31, 33, 34, 36, 37, exploring the potential interaction (especially between pancreatic beta cells and immune cell types) and outcomes upon modulation of this signaling axis could be a promising strategy to find new therapies to treat T1DM. However, further studies are needed to dissect the factors involved in this paracrine communication, in order to ensure that the outcome of modulating the pathway will be beneficial/anti-inflammatory. To sum up, modulation of *PTGS2/COX2*-PGE_2_ signaling pathway may be a promising strategy to treat different inflammation-related pathologies. However, it is vital to determine the role of PGE_2_ in each individual context, since depending on the main PTGERs/EPs mediating the effects, PGE_2_ may lead to very distinct phenotypes. In the case of T1DM, since preliminary research performed up to date has shown promising outcomes when targeting this pathway, it is conceivable that new cellular mechanisms will be revealed in the future, which could be targeted in addition to the canonical pathways with therapeutic purposes.

## References

[1] Eizirik DL, Colli ML, Ortis F (2009). The role of inflammation in insulitis and beta-cell loss in type 1 diabetes. Nat Rev Endocrinol.

[2] Cobo-Vuilleumier N, Gauthier BR (2020). Time for a paradigm shift in treating type 1 diabetes mellitus: coupling inflammation to islet regeneration. Metabolism.

[3] Lu J, Liu J, Li L, Lan Y, Liang Y (2020). Cytokines in type 1 diabetes: mechanisms of action and immunotherapeutic targets. Clin Transl Immunology.

[4] Arif S, Tree TI, Astill TP, Tremble JM, Bishop AJ, Dayan CM (2004). Autoreactive T cell responses show proinflammatory polarization in diabetes but a regulatory phenotype in health. J Clin Invest.

[5] Arif S, Moore F, Marks K, Bouckenooghe T, Dayan CM, Planas R (2011). Peripheral and islet interleukin-17 pathway activation characterizes human autoimmune diabetes and promotes cytokine-mediated beta-cell death. Diabetes.

[6] Cobo-Vuilleumier N, Lorenzo PI, Rodriguez NG, Herrera Gomez IG, Fuente-Martin E, Lopez-Noriega L (2018). LRH-1 agonism favours an immune-islet dialogue which protects against diabetes mellitus. Nat Commun.

[7] Eizirik DL, Sammeth M, Bouckenooghe T, Bottu G, Sisino G, Igoillo-Esteve M (2012). The human pancreatic islet transcriptome: expression of candidate genes for type 1 diabetes and the impact of pro-inflammatory cytokines. PLoS Genet.

[8] Perakakis N, Mantzoros CS (2016). Immune therapy in type 1 diabetes mellitus - Attempts to untie the Gordian knot?. Metabolism.

[9] Dubois RN, Abramson SB, Crofford L, Gupta RA, Simon LS, Van De Putte LB (1998). Cyclooxygenase in biology and disease. FASEB J.

[10] Alexanian A, Sorokin A (2017). Cyclooxygenase 2: protein-protein interactions and posttranslational modifications. Physiol Genomics.

[11] Klein T, Shephard P, Kleinert H, Komhoff M (2007). Regulation of cyclooxygenase-2 expression by cyclic AMP. Biochim Biophys Acta.

[12] Kawahara K, Hohjoh H, Inazumi T, Tsuchiya S, Sugimoto Y (2015). Prostaglandin E2-induced inflammation: Relevance of prostaglandin E receptors. Biochim Biophys Acta.

[13] Cheng H, Huang H, Guo Z, Chang Y, Li Z (2021). Role of prostaglandin E2 in tissue repair and regeneration. Theranostics.

[14] Chandrasekharan NV, Dai H, Roos KL, Evanson NK, Tomsik J, Elton TS (2002). COX-3, a cyclooxygenase-1 variant inhibited by acetaminophen and other analgesic/antipyretic drugs: cloning, structure, and expression. Proc Natl Acad Sci U S A.

[15] Shanmugam N, Figarola JL, Li Y, Swiderski PM, Rahbar S, Natarajan R (2008). Proinflammatory effects of advanced lipoxidation end products in monocytes. Diabetes.

[16] Chan MM, Moore AR (2010). Resolution of inflammation in murine autoimmune arthritis is disrupted by cyclooxygenase-2 inhibition and restored by prostaglandin E2-mediated lipoxin A4 production. J Immunol.

[17] Martin Vazquez E, Cobo-Vuilleumier N, Araujo Legido R, Marin-Canas S, Nola E, Dorronsoro A (2022). NR5A2/LRH-1 regulates the PTGS2-PGE2-PTGER1 pathway contributing to pancreatic islet survival and function. iScience.

[18] Dey I, Lejeune M, Chadee K (2006). Prostaglandin E2 receptor distribution and function in the gastrointestinal tract. Br J Pharmacol.

[19] Winnall WR, Ali U, O'Bryan MK, Hirst JJ, Whiley PA, Muir JA (2007). Constitutive expression of prostaglandin-endoperoxide synthase 2 by somatic and spermatogenic cells is responsible for prostaglandin E2 production in the adult rat testis. Biol Reprod.

[20] Harris RC, McKanna JA, Akai Y, Jacobson HR, Dubois RN, Breyer MD (1994). Cyclooxygenase-2 is associated with the macula densa of rat kidney and increases with salt restriction. J Clin Invest.

[21] Yaksh TL, Dirig DM, Conway CM, Svensson C, Luo ZD, Isakson PC (2001). The acute antihyperalgesic action of nonsteroidal, anti-inflammatory drugs and release of spinal prostaglandin E2 is mediated by the inhibition of constitutive spinal cyclooxygenase-2 (COX-2) but not COX-1. J Neurosci.

[22] Muller N, Riedel M, Schwarz MJ (2004). Psychotropic effects of COX-2 inhibitors-a possible new approach for the treatment of psychiatric disorders. Pharmacopsychiatry.

[23] Ibuki T, Matsumura K, Yamazaki Y, Nozaki T, Tanaka Y, Kobayashi S (2003). Cyclooxygenase-2 is induced in the endothelial cells throughout the central nervous system during carrageenan-induced hind paw inflammation; its possible role in hyperalgesia. J Neurochem.

[24] Minghetti L (2004). Cyclooxygenase-2 (COX-2) in inflammatory and degenerative brain diseases. J Neuropathol Exp Neurol.

[25] Poligone B, Baldwin AS (2001). Positive and negative regulation of NF-kappaB by COX-2: roles of different prostaglandins. J Biol Chem.

[26] Kellogg AP, Cheng HT, Pop-Busui R (2008). Cyclooxygenase-2 pathway as a potential therapeutic target in diabetic peripheral neuropathy. Curr Drug Targets.

[27] Gomez PF, Pillinger MH, Attur M, Marjanovic N, Dave M, Park J (2005). Resolution of inflammation: prostaglandin E2 dissociates nuclear trafficking of individual NF-kappaB subunits (p65, p50) in stimulated rheumatoid synovial fibroblasts. J Immunol.

[28] Dickman A, Ellershaw J (2004). NSAIDs: gastroprotection or selective COX-2 inhibitor?. Palliat Med.

[29] Parisien M, Lima LV, Dagostino C, El-Hachem N, Drury GL, Grant AV (2022). Acute inflammatory response via neutrophil activation protects against the development of chronic pain. Sci Transl Med.

[30] Luan B, Yoon YS, Le Lay J, Kaestner KH, Hedrick S, Montminy M (2015). CREB pathway links PGE2 signaling with macrophage polarization. Proc Natl Acad Sci U S A.

[31] Sreeramkumar V, Hons M, Punzon C, Stein JV, Sancho D, Fresno M (2016). Efficient T-cell priming and activation requires signaling through prostaglandin E2 (EP) receptors. Immunol Cell Biol.

[32] Sreeramkumar V, Fresno M, Cuesta N (2012). Prostaglandin E2 and T cells: friends or foes?. Immunol Cell Biol.

[33] Mahic M, Yaqub S, Johansson CC, Tasken K, Aandahl EM (2006). FOXP3+CD4+CD25+ adaptive regulatory T cells express cyclooxygenase-2 and suppress effector T cells by a prostaglandin E2-dependent mechanism. J Immunol.

[34] Yao C, Sakata D, Esaki Y, Li Y, Matsuoka T, Kuroiwa K (2009). Prostaglandin E2-EP4 signaling promotes immune inflammation through Th1 cell differentiation and Th17 cell expansion. Nat Med.

[35] Palla AR, Ravichandran M, Wang YX, Alexandrova L, Yang AV, Kraft P (2021). Inhibition of prostaglandin-degrading enzyme 15-PGDH rejuvenates aged muscle mass and strength. Science.

[36] Zhou L, Zhou W, Joseph AM, Chu C, Putzel GG, Fang B (2022). Group 3 innate lymphoid cells produce the growth factor HB-EGF to protect the intestine from TNF-mediated inflammation. Nat Immunol.

[37] Raucci F, Saviano A, Casillo GM, Guerra-Rodriguez M, Mansour AA, Piccolo M (2022). IL-17-induced inflammation modulates the mPGES-1/PPAR-gamma pathway in monocytes/macrophages. Br J Pharmacol.

[38] Ebstein W (2002). Invited comment on W. Ebstein: On the therapy of diabetes mellitus, in particular on the application of sodium salicylate. J Mol Med (Berl).

[39] Hundal RS, Petersen KF, Mayerson AB, Randhawa PS, Inzucchi S, Shoelson SE (2002). Mechanism by which high-dose aspirin improves glucose metabolism in type 2 diabetes. J Clin Invest.

[40] Yin MJ, Yamamoto Y, Gaynor RB (1998). The anti-inflammatory agents aspirin and salicylate inhibit the activity of I(kappa)B kinase-beta. Nature.

[41] Yuan M, Konstantopoulos N, Lee J, Hansen L, Li ZW, Karin M (2001). Reversal of obesity- and diet-induced insulin resistance with salicylates or targeted disruption of Ikkbeta. Science.

[42] Goldfine AB, Fonseca V, Jablonski KA, Pyle L, Staten MA, Shoelson SE (2010). The effects of salsalate on glycemic control in patients with type 2 diabetes: a randomized trial. Ann Intern Med.

[43] Persaud SJ, Burns CJ, Belin VD, Jones PM (2004). Glucose-induced regulation of COX-2 expression in human islets of Langerhans. Diabetes.

[44] Shanmugam N, Todorov IT, Nair I, Omori K, Reddy MA, Natarajan R (2006). Increased expression of cyclooxygenase-2 in human pancreatic islets treated with high glucose or ligands of the advanced glycation endproduct-specific receptor (AGER), and in islets from diabetic mice. Diabetologia.

[45] Oshima H, Taketo MM, Oshima M (2006). Destruction of pancreatic beta-cells by transgenic induction of prostaglandin E2 in the islets. J Biol Chem.

[46] Vennemann A, Gerstner A, Kern N, Ferreiros Bouzas N, Narumiya S, Maruyama T (2012). PTGS-2-PTGER2/4 signaling pathway partially protects from diabetogenic toxicity of streptozotocin in mice. Diabetes.

[47] Cipollone F, Iezzi A, Fazia M, Zucchelli M, Pini B, Cuccurullo C (2003). The receptor RAGE as a progression factor amplifying arachidonate-dependent inflammatory and proteolytic response in human atherosclerotic plaques: role of glycemic control. Circulation.

[48] Kislinger T, Fu C, Huber B, Qu W, Taguchi A, Du Yan S (1999). N(epsilon)-(carboxymethyl)lysine adducts of proteins are ligands for receptor for advanced glycation end products that activate cell signaling pathways and modulate gene expression. J Biol Chem.

[49] Takahashi P, Xavier DJ, Lima J, Evangelista AF, Collares CVA, Foss-Freitas MC (2022). Transcript Expression Profiles and MicroRNA Regulation Indicate an Upregulation of Processes Linked to Oxidative Stress, DNA Repair, Cell Death, and Inflammation in Type 1 Diabetes Mellitus Patients. J Diabetes Res.

[50] Litherland SA, Xie XT, Hutson AD, Wasserfall C, Whittaker DS, She JX (1999). Aberrant prostaglandin synthase 2 expression defines an antigen-presenting cell defect for insulin-dependent diabetes mellitus. J Clin Invest.

[51] Litherland SA, She JX, Schatz D, Fuller K, Hutson AD, Peng RH (2003). Aberrant monocyte prostaglandin synthase 2 (PGS2) expression in type 1 diabetes before and after disease onset. Pediatr Diabetes.

[52] Carboneau BA, Breyer RM, Gannon M (2017). Regulation of pancreatic beta-cell function and mass dynamics by prostaglandin signaling. J Cell Commun Signal.

[53] Medeiros A, Peres-Buzalaf C, Fortino Verdan F, Serezani CH (2012). Prostaglandin E2 and the suppression of phagocyte innate immune responses in different organs. Mediators Inflamm.

[54] Amior L, Srivastava R, Nano R, Bertuzzi F, Melloul D (2019). The role of Cox-2 and prostaglandin E2 receptor EP3 in pancreatic beta-cell death. FASEB J.

[55] Robertson RP, Tsai P, Little SA, Zhang HJ, Walseth TF (1987). Receptor-mediated adenylate cyclase-coupled mechanism for PGE2 inhibition of insulin secretion in HIT cells. Diabetes.

[56] Tran PO, Gleason CE, Robertson RP (2002). Inhibition of interleukin-1beta-induced COX-2 and EP3 gene expression by sodium salicylate enhances pancreatic islet beta-cell function. Diabetes.

[57] Bosma KJ, Andrei SR, Katz LS, Smith AA, Dunn JC, Ricciardi VF (2021). Pharmacological blockade of the EP3 prostaglandin E2 receptor in the setting of type 2 diabetes enhances beta-cell proliferation and identity and relieves oxidative damage. Mol Metab.

[58] Garber AJ (2011). Long-acting glucagon-like peptide 1 receptor agonists: a review of their efficacy and tolerability. Diabetes Care.

[59] Carboneau BA, Allan JA, Townsend SE, Kimple ME, Breyer RM, Gannon M (2017). Opposing effects of prostaglandin E2 receptors EP3 and EP4 on mouse and human beta-cell survival and proliferation. Mol Metab.

[60] Tuttle RL, Gill NS, Pugh W, Lee JP, Koeberlein B, Furth EE (2001). Regulation of pancreatic beta-cell growth and survival by the serine/threonine protein kinase Akt1/PKBalpha. Nat Med.

[61] Fatrai S, Elghazi L, Balcazar N, Cras-Meneur C, Krits I, Kiyokawa H (2006). Akt induces beta-cell proliferation by regulating cyclin D1, cyclin D2, and p21 levels and cyclin-dependent kinase-4 activity. Diabetes.

[62] Hamilton D, Rugg C, Davis N, Kvezereli M, Tafti BA, Busque S (2014). A preconditioning regimen with a PKCvarepsilon activator improves islet graft function in a mouse transplant model. Cell Transplant.

[63] Bai XM, Jiang H, Ding JX, Peng T, Ma J, Wang YH (2010). Prostaglandin E2 upregulates survivin expression via the EP1 receptor in hepatocellular carcinoma cells. Life Sci.

